# Sequestration of the Aβ Peptide Prevents Toxicity and Promotes Degradation In Vivo

**DOI:** 10.1371/journal.pbio.1000334

**Published:** 2010-03-16

**Authors:** Leila M. Luheshi, Wolfgang Hoyer, Teresa Pereira de Barros, Iris van Dijk Härd, Ann-Christin Brorsson, Bertil Macao, Cecilia Persson, Damian C. Crowther, David A. Lomas, Stefan Ståhl, Christopher M. Dobson, Torleif Härd

**Affiliations:** 1 Department of Chemistry, University of Cambridge, Cambridge, United Kingdom; 2 Department of Medical Biochemistry, University of Gothenburg, Gothenburg, Sweden; 3 Institute of Physical Biology, Heinrich-Heine-University, Dusseldorf, Germany; 4 The Swedish NMR Centre, University of Gothenburg, Gothenburg, Sweden; 5 Department of Genetics, University of Cambridge, Cambridge, United Kingdom; 6 Department of Medicine, Cambridge Institute for Medical Research, University of Cambridge, Cambridge, United Kingdom; 7 School of Biotechnology, AlbaNova University Center, Royal Institute of Technology (KTH), Stockholm, Sweden; 8 Department of Molecular Biology, Swedish University of Agricultural Sciences (SLU), Uppsala, Sweden; University of California Los Angeles, United States of America

## Abstract

Protein aggregation, arising from the failure of the cell to regulate the synthesis or degradation of aggregation-prone proteins, underlies many neurodegenerative disorders. However, the balance between the synthesis, clearance, and assembly of misfolded proteins into neurotoxic aggregates remains poorly understood. Here we study the effects of modulating this balance for the amyloid-beta (Aβ) peptide by using a small engineered binding protein (Z_Aβ3_) that binds with nanomolar affinity to Aβ, completely sequestering the aggregation-prone regions of the peptide and preventing its aggregation. Co-expression of Z_Aβ3_ in the brains of *Drosophila melanogaster* expressing either Aβ_42_ or the aggressive familial associated E22G variant of Aβ_42_ abolishes their neurotoxic effects. Biochemical analysis indicates that monomer Aβ binding results in degradation of the peptide in vivo. Complementary biophysical studies emphasize the dynamic nature of Aβ aggregation and reveal that Z_Aβ3_ not only inhibits the initial association of Aβ monomers into oligomers or fibrils, but also dissociates pre-formed oligomeric aggregates and, although very slowly, amyloid fibrils. Toxic effects of peptide aggregation in vivo can therefore be eliminated by sequestration of hydrophobic regions in monomeric peptides, even when these are extremely aggregation prone. Our studies also underline how a combination of in vivo and in vitro experiments provide mechanistic insight with regard to the relationship between protein aggregation and clearance and show that engineered binding proteins may provide powerful tools with which to address the physiological and pathological consequences of protein aggregation.

## Abstract 2

An engineered protein prevents aggregation of the Aβ peptide and facilitates clearance of Aβ from the brain in a fruit fly model of Alzheimer's disease.

## Author Summary

Alzheimer's disease is thought to be a result of neuronal damage caused by toxic aggregated forms of the Aβ peptide in the brain. There is no cure and existing treatments are ineffective in reversing or preventing disease progression. Here we describe a novel strategy that makes use of an engineered “Affibody” protein to study the disease and potentially combat its underlying causes. The Affibody occludes the aggregation-prone regions of Aβ peptides, preventing their aggregation into toxic forms, and it also acts to dissolve pre-formed Aβ aggregates. It is functional in vivo, as its co-expression with Aβ peptides in transgenic fruit flies prevents the neuronal damage and premature death that result from expression of Aβ peptides alone. Moreover, we show that the origin of this protection is the enhanced clearance of Aβ peptides from the brain. These findings open up new opportunities for using engineered binding proteins to probe the origins of Alzheimer's disease and potentially to develop a new class of therapeutic agents.

## Introduction

Of the neurodegenerative disorders that have been linked to protein misfolding and aggregation [1], Alzheimer's disease (AD) is the most common [2],[3]. Transgenic animal models have shown that aggregation of the Alzheimer β-peptide (Aβ) causes memory impairment [4],[5] and cognitive deficits [6] similar to those seen in patients suffering from AD. Aβ aggregation precedes neuritic changes [7], and there is a quantitative correlation between the propensities of mutant forms of Aβ to aggregate and their neurotoxicity [8]. In vitro aggregation of Aβ proceeds from the initial association of monomers into oligomeric, but still soluble, assemblies that ultimately form highly structured and insoluble amyloid fibrils [1],[9],[10],[11]. Evidence suggests that the primary neurotoxic species are the soluble oligomeric aggregates [4],[5],[12],[13] and that a fundamental building block may be dimeric Aβ species [14]. However, despite this progress, the details of Aβ aggregation in vivo, the structure of toxic aggregates, the mechanism of toxicity, and in particular, the relationship between aggregate formation and peptide clearance are not known.

We set out to investigate a novel approach to study the dynamics of Aβ aggregation in vitro and neurotoxicity or degradation in vivo by using a conformation-specific Aβ binding protein, the Z_Aβ3_ Affibody [15],[16]. Affibody molecules are engineered binding proteins, which are selected by phage display from libraries based on the three-helix Z domain [17],[18]. The Z_Aβ3_ Affibody was selected [15] to bind specifically to Aβ monomers with nanomolar affinity (dissociation constant K_d_≈17 nM) [16]. It forms a disulfide-linked dimer to which Aβ binds and folds by induced fit [19] into a hairpin conformation such that its two aggregation-prone hydrophobic faces become buried within a tunnel-like cavity in the Z_Aβ3_ dimer [16],[19]. The specificity and well-characterized structural features of Z_Aβ3_ binding to Aβ make it an ideal candidate for studying the effects of Aβ monomer binding in vivo. We find that the presence of the Affibody molecule, achieved by co-expression, can eliminate Aβ neurotoxicity in a fruit fly (*Drosophila melanogaster*) model of AD [20],[21], and we used biochemical and biophysical experiments to identify the molecular mechanism by which this process occurs.

## Results/Discussion

### Elimination of Aβ Neurotoxicity In Vivo

We first generated *Drosophila* strains transgenic for Z_Aβ3_. As Z_Aβ3_ is most effective in binding Aβ when it is in its dimeric form, we also generated *Drosophila* in which two copies of Z_Aβ3_ are connected head-to-tail—(Z_Aβ3_)_2_—to enable the disulfide-linked dimer to form more readily. *Drosophila* transgenic for the wild-type Z domain were used as controls. These three Affibody fly lines were then each crossed with *Drosophila* transgenic for Aβ_42_, Aβ_42_ e22g [22], or Aβ_40_, and the co-expression of both transgenes together in the brain or in the eye was initiated by crossing with appropriate driver flies [20],[21].

**Figure 1:**
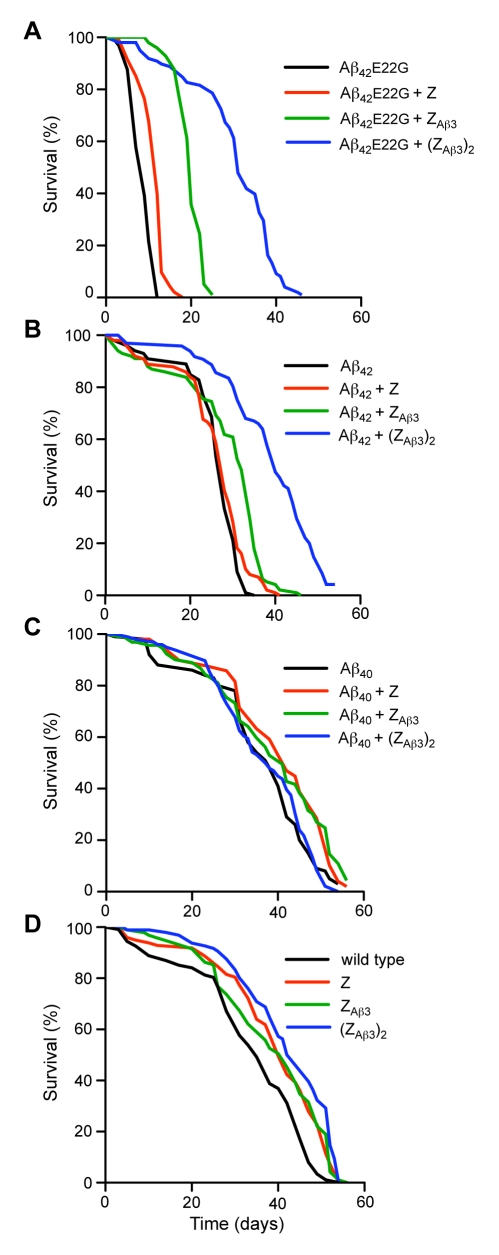
Inhibition of neurotoxicity measured as lifespan of transgenic *Drosophila*. Each curve represents 100 flies divided equally into groups of 10. Expression of all Aβ peptides and Affibody proteins was under the control of the *UAS-GAL4 system*. In these experiments, expression was driven throughout the CNS by the *elav^c155^-GAL4* driver line. Survival assays were performed to quantify the degree of neurodegeneration when each different combination of Aβ peptide and Affibody proteins or Z domain control was expressed in the CNS. (A) Aβ_42_ e22g median lifespan = 9 (±0.5) days; Aβ_42_ e22g + Z_Aβ3_ = 20 (±0.2) days, *p*<0.001 versus Aβ_42_ e22g alone; Aβ_42_ e22g + (Z_Aβ3_)_2_ = 31 (±0.8) days, *p*<0.001 versus Aβ_42_ e22g alone. (B) Aβ_42_ median lifespan = 28 (±0.4) days; Aβ_42_ + Z_Aβ3_ = 32 (±0.7) days, *p*<0.001 versus Aβ_42_ alone; Aβ_42_ + (Z_Aβ3_)_2_ = 40 (±1.2) days, *p*<0.001 versus Aβ_42_ alone. (C) Aβ_40_ median lifespan = 38 (±2); Aβ_40_ + Z_Aβ3_ = 41 (±2) days; Aβ_40_ + (Z_Aβ3_)_2_ = 38 (±2) days. (D) Control experiment: lifespan of flies expressing only the Z domain, Z_Aβ3_, or (Z_Aβ3_)_2_ and non-transgenic flies (wild-type). Median lifespan of wild-type flies = 38 (±1.8) days. Complete survival statistics are shown in Table S1.

Expression of Aβ_42_ e22g in the brain of *Drosophila* causes rapid neurodegeneration resulting in a drastic reduction in lifespan from 38 (±1.8) to 9 (±0.5) days, consistent with the findings of previous studies [8]. Co-expression of Z_Aβ3_ with Aβ_42_ e22g, however, increases the lifespan to 20 (±0.2) days. Strikingly, if the-head-to-tail dimer (Z_Aβ3_)_2_ is co-expressed with Aβ_42_ e22g, the toxic effects of the peptide are yet further reduced and the lifespan increases to 31 (±0.8) days, which is almost as long as in wild-type controls (Figure 1A, Table S1) and indicates that the neurotoxicity of Aβ has been almost entirely abolished. Co-expression of the Z domain, which has no affinity for Aβ, does not affect Aβ_42_ e22g toxicity, demonstrating that the rescue of Aβ toxicity in vivo is specific to Z_Aβ3_. Co-expression of Z_Aβ3_ with wild-type Aβ_42_ also significantly prolongs the lifespan of these flies (from 28, ±0.4, to 32, ±0.7, days). Again, the (Z_Aβ3_)_2_ head-to-tail-dimer is even more effective, completely eliminating the toxicity associated with Aβ_42_ (lifespan 40, ±1.2, days), whereas the Z domain control has no effect (Figure 1B). Expression of the less aggregation-prone Aβ_40_ has no effect on lifespan, and none of the Affibody molecules or the control significantly affected the lifespan of flies expressing Aβ_40_ or wild-type *Drosophila* (Figure 1C and 1D).

**Figure 2:**
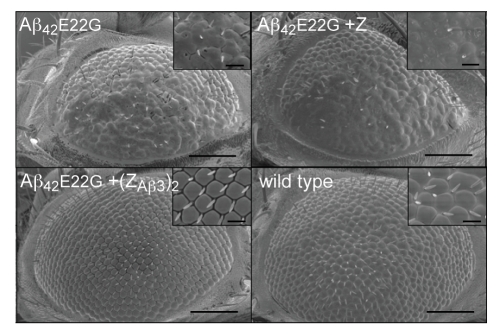
Rescue of *Drosophila* eye morphology. Scanning electron micrographs (SEM) of eyes of flies expressing Aβ_42_ e22g alone or in combination with the Z domain control or the (Z_Aβ3_)_2_ Affibody at low and high magnification. A wild-type non-transgenic fly eye is shown for comparison. Scale bar = 100 µm in main pictures and 20 µm in inserts.

The ability of (Z_Aβ3_)_2_ to abolish the toxic effects of Aβ_42_ e22g was confirmed physiologically by its ability to abolish the abnormal eye morphology associated with Aβ_42_ e22g expression in the photoreceptors in the fly (Figure 2).

### Clearance of Aβ from the *Drosophila* Brain

**Figure 3:**
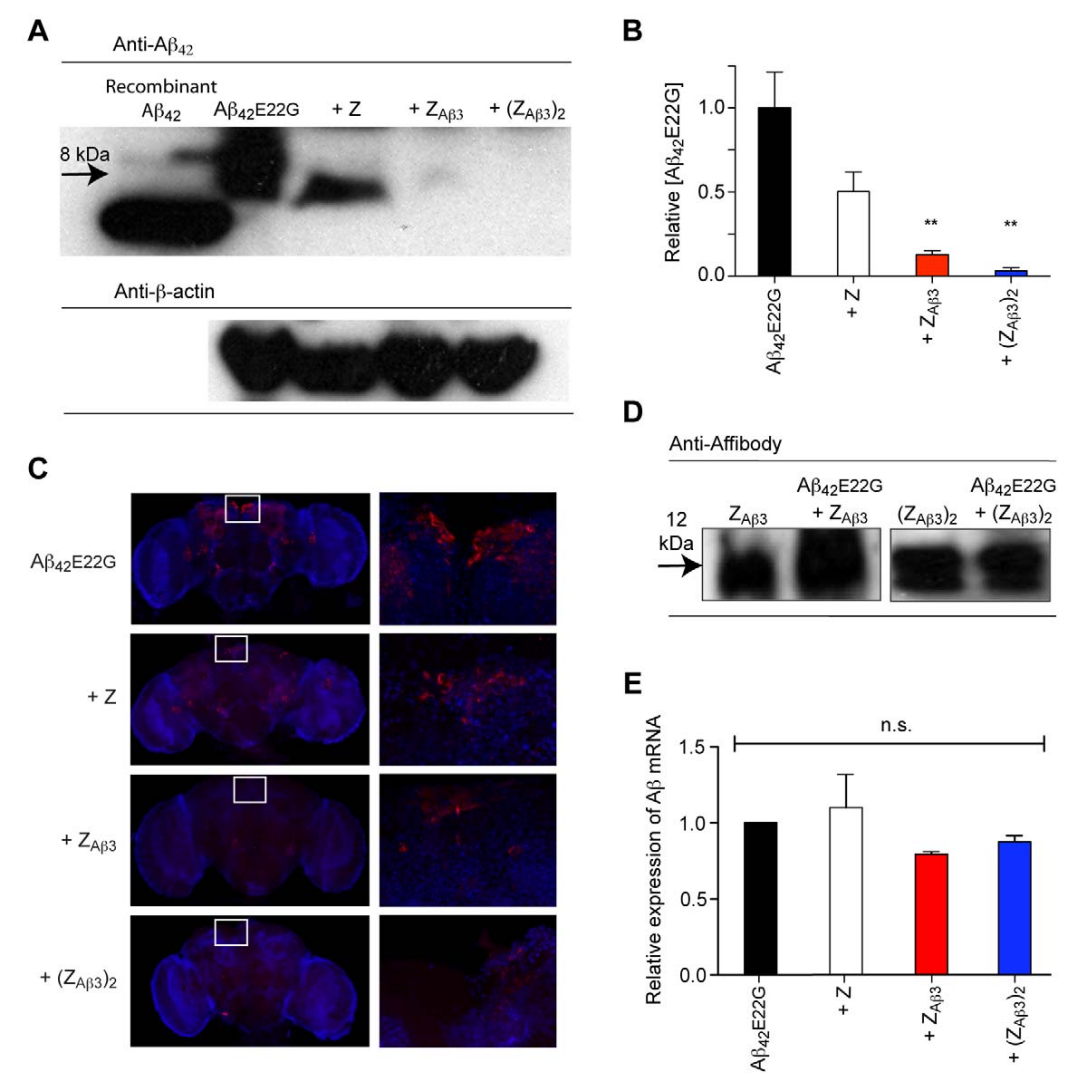
Clearance of Aβ from the *Drosophila* brain. (A) Electrophoretic (SDS PAGE) analysis of soluble Aβ in fly brain extracts. A clear Aβ immunoreactive band is seen at 8 kDa (consistent with an Aβ dimer [14]) in flies expressing Aβ_42_ e22g and flies co-expressing Aβ_42_ e22g and the Z domain. The 8 kDa Aβ immunoreactive band is absent in flies co-expressing Aβ_42_ e22g and either Z_Aβ3_ or (Z_Aβ3_)_2_. β-actin immunodetection (bottom row) served as a loading control. (B) ELISA analysis of total (soluble and insoluble) Aβ_42_ e22g concentration in the brains of flies expressing the different Affibody constructs. The levels of Aβ_42_ e22g measured in the presence of the different Affibody molecules are expressed as a percentage of the concentration in the Aβ_42_ e22g alone control. Differences between genotypes were analyzed by ANOVA and post hoc *t* tests. ** *p*<0.01. (C) Immunohistochemistry and confocal microscopy analysis of Aβ_42_ e22g aggregates in intact brains from flies expressing Aβ_42_ e22g alone or in combination with different Affibody constructs. Anti-Aβ immunostaining is shown in red, with a nuclear counterstain (TOTO-3) shown in blue. White boxes in brain images to the left are magnified to the right. Aβ immunoreactive aggregates are observed as puncta and are abundant in the brains of flies expressing Aβ_42_ e22g alone or in combination with the Z domain. Immunoreactive Aβ deposits are sparse in brains where Z_Aβ3_ is co-expressed with Aβ_42_ e22g and absent in brains where (Z_Aβ3_)_2_ is co-expressed with Aβ_42_ e22g. (D) SDS PAGE analysis of Z_Aβ3_ and (Z_Aβ3_)_2_ levels in the presence and absence of Aβ_42_ e22g. Twelve kDa anti-c-Myc immunoreactive bands (consistent with a disulfide linked Z_Aβ3_ dimer) of equal intensity are detected in Z_Aβ3_-expressing flies in the presence or absence of Aβ_42_ e22g. Twelve kDa anti-Affibody immunoreactive bands of equal intensity are also detected for the head-to-tail linked (Z_Aβ3_)_2_ dimer. (E) Quantitative RT-PCR analysis of Aβ mRNA levels in flies expressing Aβ in combination with different Affibody constructs or the Z domain control. The relative levels of Aβ mRNA detected in flies expressing Aβ_42_ e22g in combination with Z (white), Z_Aβ3_ (red), and (Z_Aβ3_)_2_ (blue) compared to that detected in flies expressing Aβ_42_ e22g alone (black) do not differ significantly (n.s., not significant).

To determine the mechanism by which Z_Aβ3_ mediates suppression of Aβ toxicity, we assessed the levels of Aβ_42_ in the brains of flies co-expressing Aβ_42_ e22g and either Z_Aβ3_, (Z_Aβ3_)_2_, or the Z domain by Western blotting. Fly brains were homogenized in 1% SDS, subjected to electrophoretic separation, and probed using an antibody against the N-terminus of Aβ, which detailed structural studies reveal remains exposed in the Aβ:Z_Aβ3_ complex [16]. SDS soluble Aβ can clearly be detected in flies expressing Aβ_42_ e22g, but it is absent in flies co-expressing Z_Aβ3_ or (Z_Aβ3_)_2_ (Figure 3A). The specificity of this effect is confirmed by the continued presence of the Aβ_42_ e22g in flies that co-express the non-binding Z domain.

The Z_Aβ3_:Aβ complex is stable in 1% SDS (B. Macao, unpublished), and Aβ remaining in complexes or in SDS insoluble aggregates in the fly brain might therefore not be detectable by Western blot. In order to address this possibility, fly brains expressing Aβ_42_ e22g with or without (Z_Aβ3_)_2_, Z_Aβ3_ or the Z domain were homogenized in 5 M GdmCl, conditions known to dissociate both Aβ aggregates and Aβ:Z_Aβ3_ complexes. The total level of Aβ_42_ e22g in these extracts was then measured by a sensitive ELISA assay (Figure 3B). Flies expressing both (Z_Aβ3_)_2_ and Aβ_42_ e22g show a 97% (±3%) reduction in the concentration of Aβ_42_ e22g compared to flies co-expressing Aβ_42_ e22g and the inert Z domain (the most appropriate control for the non-specific effects of expressing a second transgene on the levels of Aβ). Decreased Aβ_42_ e22g levels in the presence of different Affibody constructs correlate well with corresponding reduction in neurotoxicity measured by the survival assay (Figure 1).

The prevention of Aβ_42_ e22g aggregation by Z_Aβ3_ and (Z_Aβ3_)_2_ is demonstrated by immunohistochemical detection of Aβ_42_ e22g in whole mount brain preparations analyzed by confocal microscopy. Flies expressing Aβ_42_ e22g under the control of the OK107-*Gal4* driver, which drives expression in a subset of adult neurons, contain abundant deposits in the brain recognized by the anti-Aβ 6E10 antibody, whereas flies co-expressing Aβ_42_ e22g and (Z_Aβ3_)_2_ have almost no visible 6E10 immunoreactive deposits (Figure 3C). In good agreement with the results of the ELISA analysis, co-expression of Z_Aβ3_ results in a significant reduction in the burden of aggregates but does not result in their complete removal, whereas co-expression of the Z domain gives levels of Aβ deposits similar to those present in flies expressing Aβ_42_ e22g.

In order to determine whether the presence of Aβ_42_ e22g had altered the levels of Z_Aβ3_ or (Z_Aβ3_)_2_ present in the fly brain, brain homogenates were analyzed using either anti-cMyc antibodies to detect Z_Aβ3_ or anti-Affibody antibodies to detect (Z_Aβ3_)_2_; both dimeric Affibody molecules can be observed as 12 kDa dimers under non-reducing conditions. The levels of these Affibody species are not detectably altered in flies co-expressing Aβ_42_ e22g (Figure 3D) despite the marked reduction of the levels of soluble Aβ_42_ e22g (Figure 3A). While this experiment suggests that Aβ clearance could be occurring without the corresponding clearance of its binding partner Z_Aβ3_, the quantities seen by Western blot represent the equilibrium levels of these two proteins, and so would not detect any turnover in Z_Aβ3_ that may also be occurring.

We have established that the reductions in the levels of Aβ_42_ e22g peptide in the fly brain are not due to altered gene regulation in flies co-expressing Z, Z_Aβ3_, or (Z_Aβ3_)_2_, because the levels of Aβ_42_ e22g transcription are not significantly reduced in any case (Figure 3E).

In summary, Z_Aβ3_ causes a reduction in Aβ_42_ e22g levels by actively promoting its clearance from the brain. The clearance does not involve any specific antibody-mediated process, since *Drosophila* lacks an adaptive immune system [23]. In order to determine at which stages of the Aβ aggregation process the Z_Aβ3_ Affibody can intervene, we analyzed the effects of Z_Aβ3_ on the dynamic interconversion of monomeric, oligomeric, and fibrillar Aβ species in vitro.

### Inhibition of Aβ Amyloid Fibril Formation In Vitro

**Figure 4:**
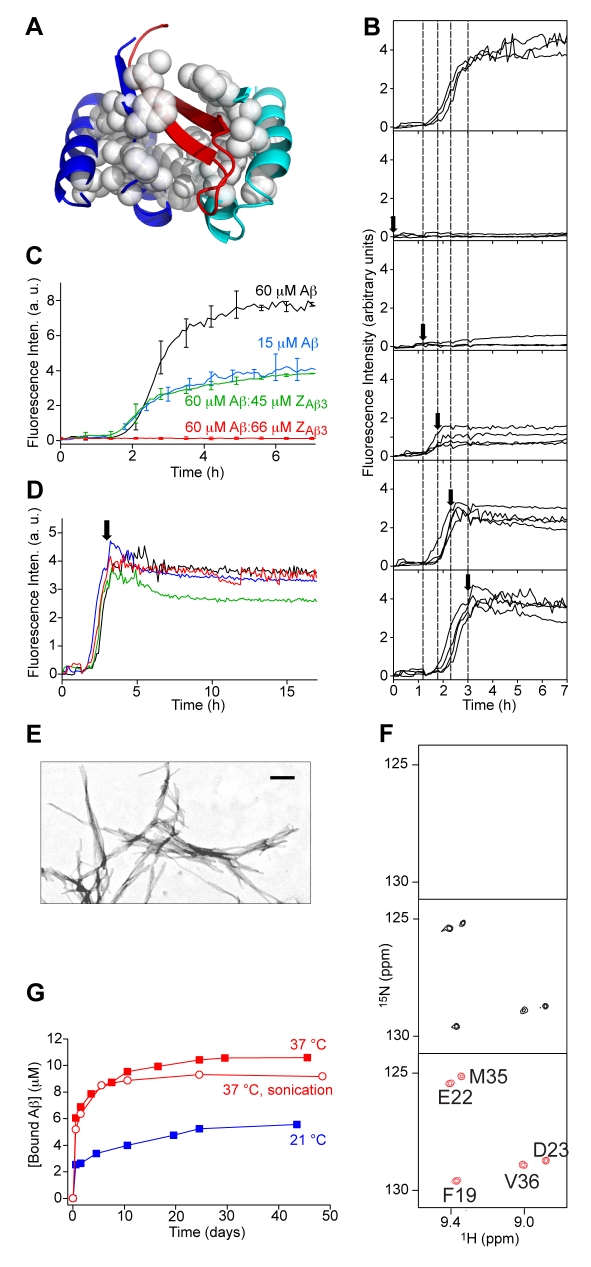
Inhibition of Aβ_40_ amyloid fibril formation. (A) Structure of the Z_Aβ3_ Affibody (blue and cyan) in complex with an Aβ_40_ hairpin (residues 16 to 40; red) [16]. White spheres represent buried nonpolar side chains (core) of Z_Aβ3_. (B–D) Kinetics of Aβ_40_ amyloid fibril formation monitored by ThT fluorescence using 30 µM Aβ_40_ with addition of 36 µM Z_Aβ3_ at different times (B and D) or using the specified concentrations of Aβ_40_ and Z_Aβ3_ (C). Time traces of three or four independent experiments are shown for each condition in (B) and (D). The average of three experiments is shown in (C) with error bars representing maximum and minimum values. Experiments in (B–D) were repeated with Aβ_42_ (Figure S3). (E) Transmission electron microscopy (TEM) of fibrils prepared for the Aβ_40_ fibril dissolution assay. Scale bar = 200 nm. (F, top) Up-field region of the ^15^N HSQC NMR spectrum of a fibril dissolution sample at 37°C starting from 300 µM ^15^N-Aβ_40_ in fibrils and (middle) 24 h after addition of 325 µM Z_Aβ3_. The Aβ_40_ backbone resonances appear as Aβ_40_ dissociates from fibrils and is captured as complex with Z_Aβ3_. For reference: the assigned spectrum of Affibody-bound monomeric Aβ_40_ (bottom) prepared directly from monomeric Aβ_40_. (G) Kinetics of Aβ_40_ fibril dissolution. The concentration of bound Aβ_40_ was calculated from the intensities of the NMR resonances compared to those of an internal ^15^N-Z_Aβ3_ standard. The experiments were carried out using recombinantly produced Aβ_40_ with an N-terminal methionine residue.

Sequestration of the hydrophobic regions of Aβ_40_ and Aβ_42_ (Figure 4A and Figure S1) allows Z_Aβ3_ to inhibit amyloid fibril formation completely, even that of the extremely aggregation-prone Aβ_42_ e22g variant, as judged by thioflavin T (ThT) fluorescence assays indicative of amyloid fibril formation (Figure 4B–D, Figure S2, and Figure S3). The addition of Z_Aβ3_ to Aβ_40_ or Aβ_42_ aggregation reactions has the same effect on the aggregation kinetics as reducing the Aβ concentration by the equivalent amount (Figure 4C and Figure S3A), demonstrating that inhibition of fibril formation occurs by sequestration of monomeric Aβ. When a molar excess of Z_Aβ3_ is added at different times during the aggregation process, it effectively inhibits all further aggregation (Figure 4B and Figure S3B), indicating that not only does Z_Aβ3_ effectively block aggregation even after its initiation, but also that monomeric Aβ is accessible for binding throughout the process of fibril formation.

### Kinetics of Amyloid Fibril Dissolution

We noted, however, during the course of the experiments that the ThT fluorescence signal tends to fall after the addition of Z_Aβ3_ at advanced stages of the fibril formation reaction, suggesting that Z_Aβ3_ may also act to reverse the aggregation process (Figure 4D and Figure S3C). To determine the kinetics of fibril dissolution by Z_Aβ3_ in vitro, we set up experiments in which Aβ_40_ monomers dissociating from pre-formed fibrils are captured by Z_Aβ3_. We used ^15^N-labelled Aβ_40_ for these experiments so that monomeric Aβ_40_ in complex with Z_Aβ3_ could be identified by solution nuclear magnetic resonance (NMR) spectroscopy at low micromolar concentrations. The large fibrillar aggregates of ^15^N-Aβ_40_ (Figure 4E) did not generate an observable NMR spectrum even after 24 h of data collection, as expected, due to slow molecular tumbling and no highly mobile residues. The addition of Z_Aβ3_, however, generated resonances from Z_Aβ3_-bound monomeric ^15^N-Aβ_40_, indicating a gradual dissolution of the fibrils (Figure 4F and Figure S4). Only a small fraction of the Aβ_40_, however, dissociates from the fibrils over the first three weeks; thereafter the dissolution process becomes very slow, even for fibrils fragmented by sonication (Figure 4G). Still, under these conditions the observed level of dissolution does not represent the equilibrium state, as the pre-formed Aβ_40_:Z_Aβ3_ complex is stable in the presence of Aβ_40_ fibrils (Figure S5). Hence, even though binding of the Z_Aβ3_ Affibody to monomeric Aβ_40_ can act to dissolve fibrils, the dissociation kinetics are too slow, at least in vitro, for dissolution to be achievable in practice under ambient conditions.

### Inhibition and Dissolution of Aβ Oligomers

**Figure 5:**
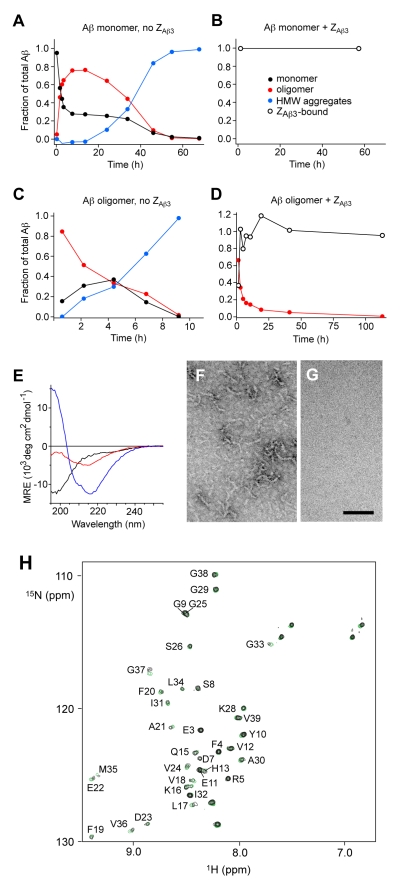
Dissolution of Aβ oligomers. (A–D) Oligomer formation (A and B; 100 µM total Aβ_42_) and oligomer dissolution (C and D; 20 µM total Aβ_42_) monitored by SEC in the absence or presence of 1.2-fold excess of Z_Aβ3_. SEC elution profiles were integrated and normalized (see Figure S6 and Materials and Methods). The fraction of high molecular weight (HMW) aggregates was calculated as the difference between unity and the sum of the monomer and oligomer fractions. (E) Normalized CD spectra (MRE, mean residue elliptictiy) of monomeric Aβ_42_ (black), oligomers (red), and fibrils (blue). β-sheet secondary structure is identified by a distinct minimum at ∼215 nm in the spectrum. (F,G) TEM images of oligomeric Aβ_42_ solutions after isolation and at the endpoint of the dissolution experiment. Scale bar = 100 nm. (H) ^15^N HSQC NMR spectrum of an Aβ_42_ oligomer sample, which has dissociated as a result of the sequestering of monomeric Aβ_42_ by Z_Aβ3_ (black). Starting from 11 µM ^15^N-Aβ_42_ in oligomeric form (such as shown in F), this spectrum was recorded 2 days after the addition of 13 µM Z_Aβ3_. The fraction of Aβ_42_ bound to Z_Aβ3_ after 5 days of incubation was estimated by NMR to be 92% (±9%). A spectrum of Z_Aβ3_:Aβ_42_ prepared from monomer solutions is shown for reference (green). The experiments were carried out using recombinantly produced Aβ_40_ or Aβ_42_ with N-terminal methionines.

In order to determine the critical issue of whether or not Z_Aβ3_ can prevent the formation of smaller Aβ aggregates (oligomers), we examined their formation in vitro by size exclusion chromatography (SEC) in the presence and absence of Z_Aβ3_ (Figure 5A to 5D and Figure S6). Oligomeric species [24] appear within hours in solutions of Aβ_42_, prepared by dilution from alkaline conditions [25], where the monomeric species is initially dominant. The partitioning between monomeric and oligomeric Aβ then reaches an interim steady state after ∼10 h before the onset of the formation of amyloid fibrils (Figure 5A). By contrast, in the presence of the Z_Aβ3_, oligomer formation is completely inhibited (Figure 5B), a result that can be attributed to the sequestration of Aβ_42_ within the complex formed with the Affibody.

Isolated Aβ_42_ oligomers contain elements of well-defined β-sheet structure as measured by circular dichroism (CD), but the β-sheet content is lower than in mature fibrils (Figure 5E). Their stability is also lower as isolated oligomers dissociate into monomers and convert into amyloid fibrils (Figure 5C). Addition of the Z_Aβ3_ Affibody results in dissolution of the oligomers after a few days (Figure 5D, 5F, and 5G and Figure S7). This is because binding of monomeric Aβ acts to shift the dynamic monomer-oligomer equilibrium such that the oligomer population is reduced, and NMR (Figure 5H) and SEC analyses (Figure S6) consequently also reveal monomeric Aβ_42_ in complex with Z_Aβ3_.

### Conclusion

The presence of the Z_Aβ3_ Affibody in vivo results in the effective inhibition of Aβ toxicity and the promotion of Aβ degradation. These effects can be attributed to the ability of the Z_Aβ3_ Affibody to act in three distinct ways on the Aβ aggregation process. First, monomeric Aβ will be sequestered by Z_Aβ3_, the result of which is that toxic Aβ aggregates will not be able to form in the brain. Second, if Aβ aggregation were to occur, it can be slowed, halted, and even reversed by the action of Z_Aβ3_ on the dynamic Aβ monomer-aggregate equilibria. Furthermore, the presence of Z_Aβ3_ not only prevents or reverses Aβ aggregate formation, it also promotes clearance from the brain. We envisage that this could occur either by intracellular lysosomal or proteasomal degradation, or alternatively by the secretion and uptake by phagocytic cells of the Z_Aβ3_:Aβ complex.

The results furthermore demonstrate how engineered binding proteins, such as Affibody molecules, that target specific protein conformations can be used to gain important insights into the dynamics of the Aβ aggregation process and its toxic consequences both in vivo and in vitro.

## Materials and Methods

### Fly Genetics

*Drosophila melanogaster* transgenic for Aβ_40_, Aβ_42_, and Aβ_42_ e22g have been described previously [20]. *Drosophila* transgenic for the Z domain, Z_Aβ3_, and the (Z_Aβ3_)_2_ head-to-tail dimer were created by standard p element mediated germ line transformation using pUAST (Brand and Perrimon) as the expression vector. Affibody cDNA was inserted into the multiple cloning site of pUAST using EcoR1 and Xho1, except for (Z_Aβ3_)_2_, which was cloned between EcoR1 and Xba1 sites. Each transgene was preceded by the same secretion signal peptide (MASKVSILLLLTVHLLAAQTFAQ), derived from the *Drosophila* necrotic gene, in order to target its expression to the secretory pathway. Transgenes were injected into w1118 embryos.

*Drosophila* transgenic for Aβ_40_, Aβ_42_, and Aβ_42_ e22g were each crossed with *Drosophila* transgenic for Z, Z_Aβ3_, and (Z_Aβ3_)_2_ to create stable double transgenic stocks. Expression of the transgenes was achieved using the UAS-Gal4 system. UAS-Tg flies were crossed with flies expressing Gal4 under the control of either a neuronal promoter (elavc155 or OK107) or eye specific promoter (gmr). All fly crosses were maintained on standard cornmeal/agar fly food in humidified incubators. Crosses to generate flies expressing Affibody molecules or Aβ were performed at 29°C.

### Survival Assays

Survival assays were performed as described previously [20]. Briefly, 100 flies of each genotype were collected, divided into tubes of 10 flies, and kept at 29°C. The number of live flies was counted every 2–3 days and recorded. Survival curves were calculated using the Kaplan-Meier method, and differences between genotypes were assessed using the log-rank test.

### Rough Eye Phenotype

Transgenes were expressed in the eye by crossing with *gmr-Gal4* flies. Crosses were performed at 29°C. Flies were collected on the day of eclosion and sputter coated using 20 nM of Au/Pd in a Polaron E5000. SEM images were collected using a Philips XL30 Microscope.

### Protein Extraction and Western Blotting

Fifty flies were snap frozen in liquid nitrogen and decapitated for each genotype. Fly heads were homogenized in PBS/1% SDS containing protease inhibitors (Complete, Roche Applied Science, UK). Homogenates were then centrifuged at 12,100 g for 1 min to remove insoluble material, and the supernatants were collected for analysis. Protein concentration in each supernatant was determined using the DC Protein Assay (Biorad). Equal quantities of protein for each genotype were loaded on to 4%–12% Bis/Tris SDS PAGE gels (Invitrogen) for detection of Affibody molecules and 4%–12% Tris/glycine SDS PAGE gels (Invitrogen) for detection of Aβ. Electrophoresis was performed under non-reducing conditions, and protein was transferred to nitrocellulose membranes for Western blotting. Z_Aβ3_ was detected using a mouse monoclonal anti-c-Myc antibody (clone 9E10, Abcam), and (Z_Aβ3_)_2_ was detected using a goat anti-Affibody antibody (Abcam). Aβ was detected using a mouse monoclonal anti-Aβ antibody directed against the N terminus of Aβ (6E10, Signet). All blots were developed using Supersignal West Femto Maximum Sensitivity ECL substrate (Pierce).

### Total Aβ ELISA

Heads from flies expressing Aβ_42_ e22g with or without Affibody domains were subjected to mechanical homogenization in 5 M GdmCl, 50 mM Hepes, and 5 mM EDTA followed by 4 min of sonication in a water bath. Homogenates were centrifuged for 7 min at 12,100 g to pellet any GdmCl insoluble material. Supernatants were diluted in 50 mM Hepes and 5 mM EDTA with protease inhibitors to a final concentration of 1 M GdmCl. A sandwich ELISA was performed on the supernatants using biotinylated 6E10 (Signet) and a C terminal Aβ_x-42_-specific antibody 21F12 (kind gift of D. Schenk, Elan). Protein levels were measured using a Sector Imager (Meso Scale Discovery) and normalized to a percentage of the level obtained for flies expressing Aβ_42_ e22g alone.

### Immunohistochemistry

Flies of all genotypes were crossed with OK107-*Gal4* flies (Bloomington Stock No. 854) to drive expression in a subset of neurons that includes, but is not limited to, the mushroom bodies. For each genotype fly brains were dissected in PBS with 0.05% Triton X-100 and fixed in 4% paraformaldehyde for 1 h at room temperature. The brains were then washed three times in PBS/0.05% Triton X-100 and blocked in 5% w/v bovine serum albumin in PBS for 1 h at room temperature. Fly brains were incubated overnight in mouse anti-Aβ (6E10, Signet) diluted 1∶1000 in blocking buffer. After three further washes in PBS/0.05% Triton X-100, brains were then incubated in goat anti-mouse IgG Alexa 546 (Invitrogen) and counterstained with TOTO-3 (Invitrogen) to detect nuclei before mounting in Vectashield (Vectorlabs) anti-fade mounting medium.

### Confocal Microscopy

Confocal serial scanning images were acquired at 2 or 4 µm intervals (for high magnification and low magnification images, respectively) using a Nikon Eclipse C1si on Nikon E90i upright stand (Nikon). The image stacks were projected using ImageJ (version 1.42k), and the resulting composite images were processed using Photoshop CS4 software (Adobe Systems).

### Transcription Assay

Concentrations of mRNA were determined using quantitative real time PCR (RT-PCR). Twenty-five flies per genotype were collected and snap frozen in liquid N_2_. RNA was extracted from each group of 25 fly heads using TriZol followed by DNAse treatment to remove residual genomic DNA and reverse transcription to produce cDNA. Each sample was subjected to two separate quantitative PCR reactions to detect Aβ mRNA and the control gene Actin5c. Real time amplification of cDNA was monitored using SYBR Green fluorescence in a Bio-Rad iQ Cycler.

### Protein Samples for Biophysical Analysis

Z_Aβ3_ was produced in *Escherichia coli* and purified as described elsewhere [16]. Aβ peptides were obtained from a commercial source (rpeptide, Bogart, GA, USA), synthesized in-house, or produced (with an N-terminal methionine) by recombinant co-expression of Aβ and Z_Aβ3_ in *E. coli* [26]. Experiments were carried out in 20 mM sodium phosphate, 50 mM NaCl, except for the NMR experiments where NaCl was not included, and pH 7.2. 10 µM ThT was added prior to fluorescence measurements.

### Aβ Fibril Formation

Fibril formation assays were carried out as described previously [16]. TEM images were obtained using a LEO 912 AB Omega microscope. CD spectra were recorded on a JASCO J-810 spectropolarimeter.

### Aβ Fibril Dissolution

Fibrils were prepared from Aβ_40_ at a concentration of 100 µM with the same set-up and conditions as for the fibril formation assays, but in the absence of ThT. After 3 days of incubation at 37°C, fibrils were isolated by centrifugation at 16,000 g. To remove any residual soluble peptide, fibrils were washed by resuspension in buffer F [20 mM sodium phosphate, pH 7.2, 0.1% sodium azide, complete protease inhibitor (Roche; at the concentration recommended by the manufacturer)], followed by centrifugation. Fibrils were resuspended in buffer F supplemented with 10% D_2_O to a final concentration of 300 µM Aβ_40_ and investigated by ^15^N HSQC NMR with 24 h of data collection on a Varian Inova 900 MHz NMR spectrometer (equipped with a cryogenic probe) or on a Varian Inova 800 MHz spectrometer. The intensity of resonances originating from bound Aβ_40_ detected in the presence of 325 µM of unlabeled Z_Aβ3_ was followed over time by recording a series of 24 h ^15^N HSQC NMR spectra. Five µM of ^15^N-Z_Aβ3_ served as an internal concentration reference, assuming identical NMR-sensitivities of the intense resonances of the three C-terminal residues of bound Aβ_40_ and free Z_Aβ3_. Sonication was achieved by placing the NMR tube with the fibril sample into a Misonix water bath sonicator for 2 min before acquisition of NMR data.

### Aβ Oligomer Formation and Dissolution

Oligomer formation was induced by adjusting the pH of alkaline (pH∼10.5) solutions of Aβ_42_ (concentration ≤100 µM) in 20 mM sodium phosphate and 50 mM sodium chloride to pH 7.2 (with 1 M HCl) [25]. The samples were incubated at 21°C and oligomer formation was monitored with SEC and ThT fluorescence. Fifty µl (for analytical runs) or 1 ml (for preparative oligomer isolation) aliquots were injected onto an ÄKTA Explorer system (GE Healthcare, Uppsala, Sweden) equipped with a Superdex 75 10/300 column, and the elution was monitored by UV absorbance at 220 nm. Preparative oligomer isolation was carried out 4–20 h after induction of oligomer formation and yielded oligomer solutions at 10–20 µM total Aβ_42_ concentration. The elution volumes of the Z_Aβ3_:Aβ_42_ complex and free Z_Aβ3_ were determined in separate runs of the isolated complex or free Affibody, respectively, and conformed to previous SEC studies [19]. The amounts of Aβ_42_ in the monomeric, oligomeric, or Z_Aβ3_-bound fraction were determined from the elution peak areas obtained by integration using the Unicorn software provided with the chromatography system. The data were normalized by setting to unity the sum of the oligomer and monomer peak areas in the first SEC profiles (at *t* = 0.2 h for oligomer formation in Figure S6A, and at *t* = 0.5 h for oligomer dissolution in Figure S6C). The fraction of high molecular weight aggregates that did not enter the column bed was calculated as the difference between unity and the sum of the monomer and oligomer fractions. The fraction of Z_Aβ3_-bound Aβ_42_ shown in Figure 5D was obtained by comparison of the integrated Z_Aβ3_:Aβ_42_/free Z_Aβ3_ peak area with those obtained in calibration runs of free Z_Aβ3_ (set to 0) and Z_Aβ3_:Aβ_42_ complex (set to 1) using the same protein concentrations as in the dissolution experiment. The fraction of Aβ_42_ bound to Z_Aβ3_ was determined by ^15^N HSQC NMR employing an internal concentration standard.

## Supporting Information

Figure S1 **The Z_Aβ3_-binding modes of Aβ_40_ and Aβ_42_ are identical.** ^15^N-HSQC NMR spectra of Aβ_40_ (red) and Aβ_42_ (blue) in the Z_Aβ3_-bound state. The backbone amide resonances for residues 1 to 39, including all those assigned to the β-hairpin in the core of the complex, coincide. This demonstrates that the mode of binding is identical for Aβ_40_ and Aβ_42_. Buffer, 20 mM sodium phosphate, pH 7.2. Temperature, 21°C.
(0.23 MB TIF)

Figure S2 **Z_Aβ3_ inhibits fibril formation of Aβ_42_ and Aβ_42_E22G.** (A,B) Aggregation time courses of Aβ_42_ and Aβ_42_E22G in the absence (blue) and presence (green and red) of increasing molar equivalents of Z_Aβ3_ monitored by thioflavin T fluorescence. (C) TEM images of the end stage aggregates of Aβ_42_ in the absence (left) or presence (right) of an equivalent amount of Z_Aβ3_. Scale bar = 200 nm. Peptides were purchased from Bachem and dissolved in 5 mM NaOH followed by filtration using Centricon YM-10. Solutions were then divided into aliquots and lyophilized. The quantity of peptide in the aliquots was determined by amino acid analysis. Aggregation assay samples in (A) and (B) contained 40 µl of 20 µM Aβ_42_ or 10 µM Aβ_42_ e22g in 50 mM Na-phosphate, pH 7.4, and 10 µM Thioflavin T, supplemented with the indicated amount of disulfide linked Z_Aβ3_. Samples were incubated at 37°C and data points were recorded every 4 min (Aβ_42_) or 2 min (Aβ_42_ e22g) with 10 s of orbital shaking preceding the measurement using a FLUOstar OPTIMA reader (BMG) equipped with 440 nm excitation and 480 nm emission filters. Samples analyzed by TEM (in C) were applied to formvar/carbon coated copper grids, stained with 2% (w/v) uranyl acetate, and viewed in a Philips CEM100 transmission electron microscope.
(0.80 MB TIF)

Figure S3 **The Z_Aβ3_ Affibody inhibits fibril formation of Aβ_42_ by sequestration of monomeric peptide.** (A) Aggregation time course of Aβ_42_ at the specified concentrations of Aβ_42_ and Z_Aβ3_. Averages of four experiments are shown with error bars representing estimated standard deviations. (B) Aggregation time course of Aβ_42_ using 30 µM Aβ_42_ without (black) or with addition of 36 µM Z_Aβ3_ at the times indicated by the arrows. Averages of four experiments are shown with error bars representing estimated standard deviations. (C) The four individual time traces resulting in the magenta time course in (B). Aggregation was monitored by thioflavin T fluorescence on a FarCyte reader (Tecan) equipped with 440 nm excitation and 480 nm emission filters. The samples contained ∼100 µl of the peptide/protein solution in 20 mM Na-phosphate (pH 7.2), 50 mM NaCl, and 10 µM thioflavin T. Plates were sealed with polyolefin tape (Nunc) and incubated at 37°C. Data points were recorded every 5 min with 2 min of linear shaking before the measurement. The experiments were carried out using recombinantly produced Aβ_42_ with an N-terminal methionine.
(0.31 MB TIF)

Figure S4 **Dissolution of ^15^N-Aβ_40_ from fibrils by Z_Aβ3_ monitored by NMR.** ^15^N HSQC NMR spectrum of a fibril dissolution sample (black), starting from 300 µM ^15^N-Aβ_40_ in fibrils, recorded during the first 24 h after addition of 325 µM Z_Aβ3_ and 5 µM ^15^N-Z_Aβ3_. For reference, the spectra of bound Aβ_40_ (red; assigned) and free Z_Aβ3_ (green) are shown. (The spectrum of fibrillar Aβ_40_ before Z_Aβ3_ addition shows no resonances at this contour levelling). Buffer, 20 mM sodium phosphate, pH 7.2. Temperature, 37°C. Recombinantly produced Aβ_40_ with an N-terminal methionine was used.
(0.32 MB TIF)

Figure S5 **Stability of the Aβ_40_:Z_Aβ3_ complex in the presence of Aβ_40_ amyloid fibrils.** (A) ^15^N-HSQC NMR spectrum of 100 µM ^15^N-Z_Aβ3_ bound to 100 µM unlabeled Aβ_40_ before addition and (B) after addition of 100 µM ^15^N-Aβ_40_ in amyloid fibrils and incubation for 5 days at 37°C. Buffer, 20 mM sodium phosphate, pH 7.2, 0.1% sodium azide. Fibrillar ^15^N-Aβ_40_ is not detected by solution NMR because of its large size, for which slow tumbling results in line broadening. The spectrum of ^15^N-Z_Aβ3_ in the bound state (A) is retained in (B), and resonances of ^15^N-Z_Aβ3_ in the free state do not appear. This demonstrates that Aβ_40_ does not leave the complex to be incorporated into the fibrils, i.e. the complex is stable in the presence of Aβ_40_ amyloid fibrils. Moreover, resonances of ^15^N-Aβ_40_ bound to Z_Aβ3_ do not appear in (B), i.e. ^15^N-Aβ_40_ monomers do not dissociate from the fibrils to exchange with unlabeled Aβ_40_ monomers in the Z_Aβ3_ complex. This finding is in agreement with the high kinetic stability of Aβ amyloid fibrils reported in this study. The lifetime of the Aβ_40_:Z_Aβ3_ complex was determined as 2.6 (±0.3) h at 21°C. Dissociation of the complex cannot therefore be rate-limiting in this experiment. Lifetime determination was carried out by successive recording of the ^15^N-HSQC NMR spectrum of ^15^N-Z_Aβ3_:^15^N-Aβ_40_ complex after addition of an excess of unlabeled Z_Aβ3_ and monitoring the decrease in the intensity of the resonances assigned to bound ^15^N-Z_Aβ3_. Recombinantly produced Aβ_40_ with an N-terminal methionine was used.
(0.20 MB TIF)

Figure S6 **Aβ_42_ oligomer formation and dissolution analyzed by SEC.** Elution volumes of monomeric and oligomeric Aβ_42_, free Z_Aβ3_ Affibody, and the Z_Aβ3_:Aβ_42_ complex on a Superdex 75 10/300 column, with a nominal resolution of 3,000 to 70,000 Da, are indicated. Aβ_42_ oligomers elute at the void volume (8.3 ml) and Aβ_42_ fibrils cannot enter the column. (A) A solution of 100 µM Aβ_42_ was incubated without stirring at 20°C. SEC analysis of samples removed at different times reveals the decrease in concentration of monomeric Aβ_42_ with time and the transient formation of oligomeric species, followed by formation of HMW aggregates (fibrils). (B) Analysis of an equivalent Aβ_42_ solution also containing a 1.2-fold excess of the Z_Aβ3_ Affibody shows that the Z_Aβ3_:Aβ_42_ remains stable without oligomer or HMW aggregate formation. (C,D) Oligomer dissolution: isolated oligomer Aβ_42_ fractions isolated subjected to a second incubation followed by SEC analysis. In the absence of Z_Aβ3_ (C), these dissolve on a timescale of several hours and monomeric Aβ_42_ appears transiently prior to fibril formation. Oligomer dissolution in the presence of an 1.2-fold excess of Z_Aβ3_ (D) results in Z_Aβ3_:Aβ_42_ complex formation manifested in a small but significant shift in the elution volume of the Z_Aβ3_ Affibody. Recombinantly produced Aβ_42_ with an N-terminal methionine was used.
(0.18 MB TIF)

Figure S7 **Aβ_42_ oligomer dissolution analyzed by ThT fluorescence.** Aβ_42_ oligomer fractions were isolated by SEC and incubated at 20°C. The initial fluorescence (red bar) associated with ThT binding to oligomeric Aβ_42_ increases upon formation of fibrils (blue) or decreases as oligomers dissolve in the presence of an excess of Z_Aβ3_ (grey). ThT fluorescence was recorded on a Varian Cary Eclipse spectrofluorometer at 480 nm, with excitation at 446 nm. Samples were diluted to final Aβ_42_ concentrations of 1 µM into 20 mM sodium phosphate, 50 mM NaCl, pH 7.2, supplemented with 10 µM ThT. The intensity of the fibril sample was set to unity. Error bars give the estimated standard deviation of four independent oligomer dissolution experiments. Recombinantly produced Aβ_42_ with an N-terminal methionine was used.
(0.09 MB TIF)

Table S1 **Transgenic fly survival (median life span).**
(0.05 MB PDF)

## Abbreviations

Aβ: amyloid-β peptide
AD: Alzheimer's disease
CD: circular dichroism
ELISA: enzyme-linked immunosorbant assay
GdmCl: guanidinium chloride
HSQC: hetero-nuclear single quantum coherence
NMR: nuclear magnetic resonance
PCR: polymerase chain reaction
RT-PCR: real time PCR
SDS: sodium dodecyl sulfate
SDS-PAGE: SDS-polyacrylamide electrophoresis
SEC: size exclusion chromatography
ThT: thioflavin T
Z_Aβ3_: Aβ-binding Affibody molecule
