# Supplementary figures and images for: Sequestration of the Aβ Peptide Prevents Toxicity and Promotes Degradation In Vivo
Source: PLoS Biol. 2010 Mar 16;8(3):e1000334. doi: 10.1371/journal.pbio.1000334 (PMC2838747; doi:10.1371/journal.pbio.1000334)

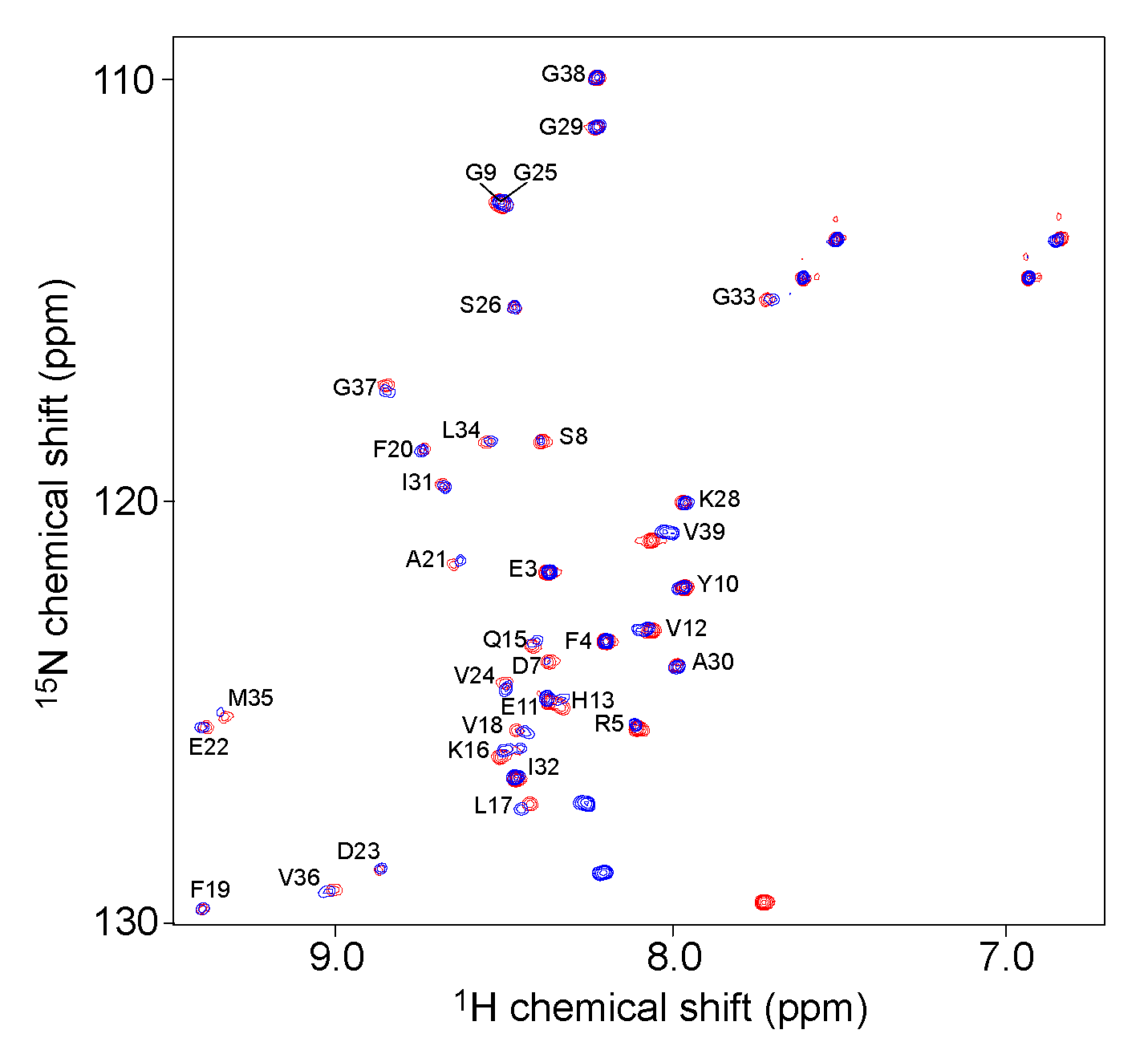

Supplement: Figure S1 — The ZAβ3-binding modes of Aβ40 and Aβ42 are identical. 15N-HSQC NMR spectra of Aβ40 (red) and Aβ42 (blue) in the ZAβ3-bound state. The backbone amide resonances for residues 1 to 39, including all those assigned to the β-hairpin in the core of the complex, coincide. This demonstrates that the mode of binding is identical for Aβ40 and Aβ42. Buffer, 20 mM sodium phosphate, pH 7.2. Temperature, 21°C. (0.23 MB TIF) [file pbio.1000334.s001.tif]

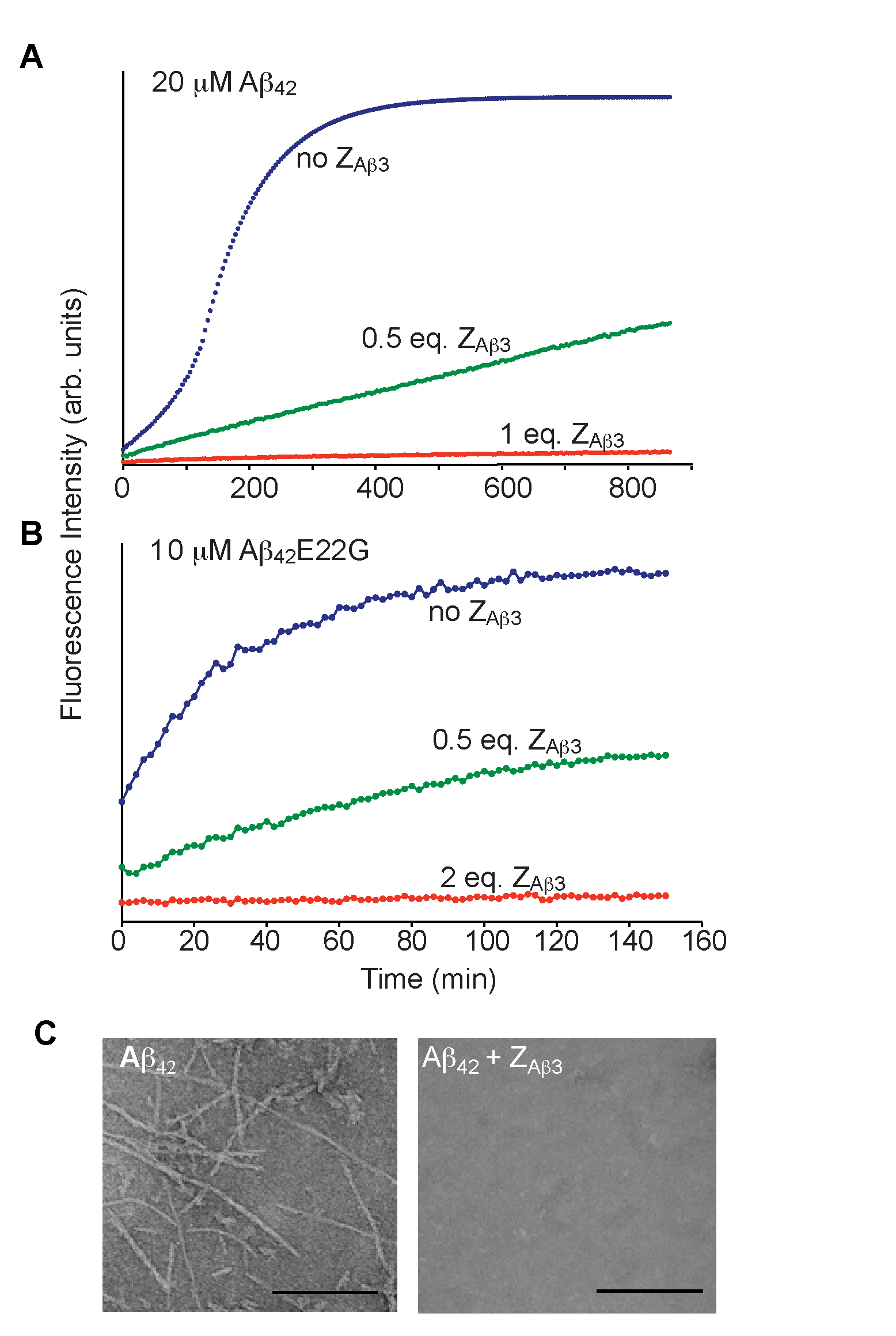

Supplement: Figure S2 — ZAβ3 inhibits fibril formation of Aβ42 and Aβ42E22G. (A,B) Aggregation time courses of Aβ42 and Aβ42E22G in the absence (blue) and presence (green and red) of increasing molar equivalents of ZAβ3 monitored by thioflavin T fluorescence. (C) TEM images of the end stage aggregates of Aβ42 in the absence (left) or presence (right) of an equivalent amount of ZAβ3. Scale bar = 200 nm. Peptides were purchased from Bachem and dissolved in 5 mM NaOH followed by filtration using Centricon YM-10. Solutions were then divided into aliquots and lyophilized. The quantity of peptide in the aliquots was determined by amino acid analysis. Aggregation assay samples in (A) and (B) contained 40 µl of 20 µM Aβ42 or 10 µM Aβ42 e22g in 50 mM Na-phosphate, pH 7.4, and 10 µM Thioflavin T, supplemented with the indicated amount of disulfide linked ZAβ3. Samples were incubated at 37°C and data points were recorded every 4 min (Aβ42) or 2 min (Aβ42 e22g) with 10 s of orbital shaking preceding the measurement using a FLUOstar OPTIMA reader (BMG) equipped with 440 nm excitation and 480 nm emission filters. Samples analyzed by TEM (in C) were applied to formvar/carbon coated copper grids, stained with 2% (w/v) uranyl acetate, and viewed in a Philips CEM100 transmission electron microscope. (0.80 MB TIF) [file pbio.1000334.s002.tif]

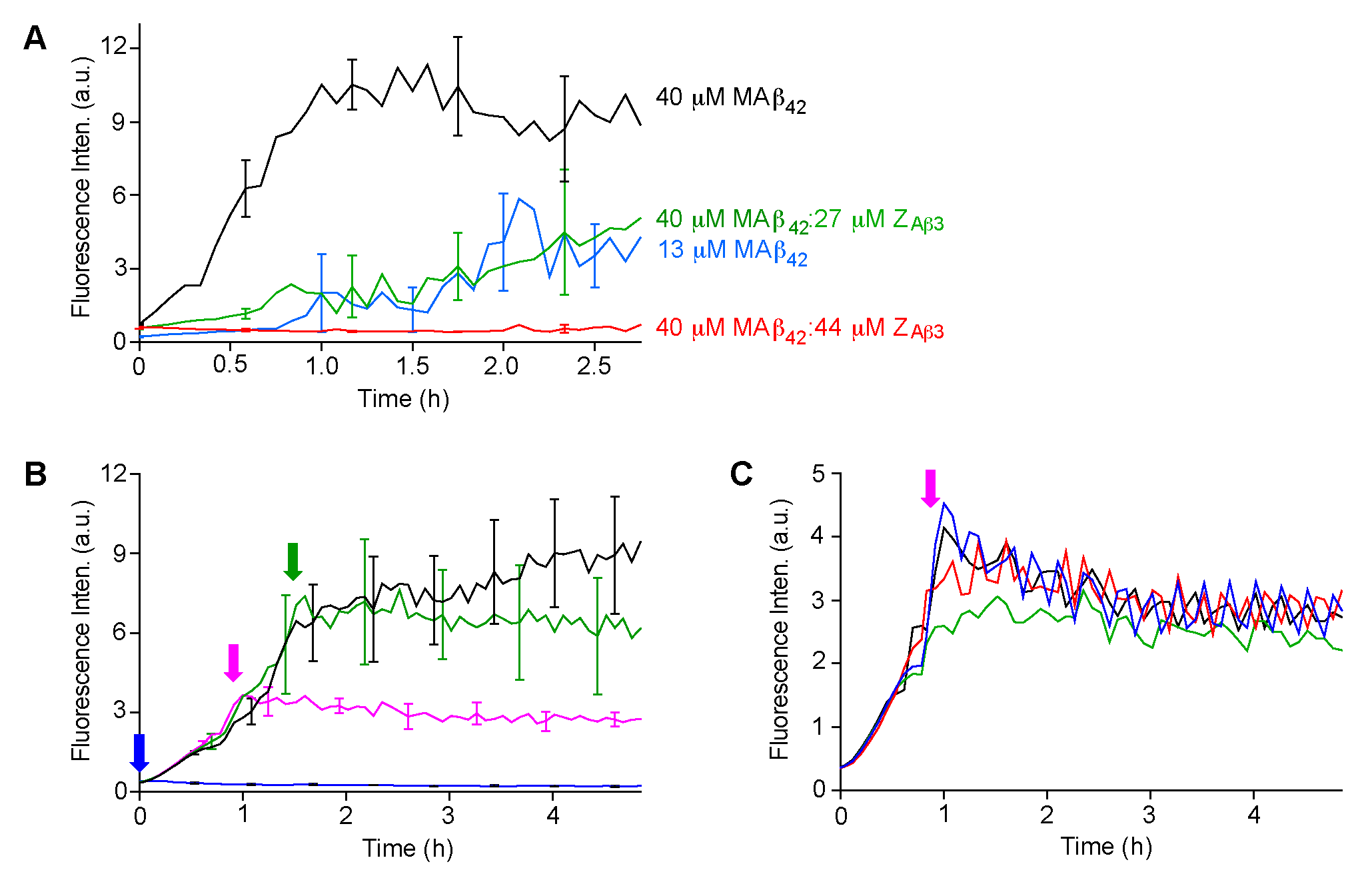

Supplement: Figure S3 — The ZAβ3 Affibody inhibits fibril formation of Aβ42 by sequestration of monomeric peptide. (A) Aggregation time course of Aβ42 at the specified concentrations of Aβ42 and ZAβ3. Averages of four experiments are shown with error bars representing estimated standard deviations. (B) Aggregation time course of Aβ42 using 30 µM Aβ42 without (black) or with addition of 36 µM ZAβ3 at the times indicated by the arrows. Averages of four experiments are shown with error bars representing estimated standard deviations. (C) The four individual time traces resulting in the magenta time course in (B). Aggregation was monitored by thioflavin T fluorescence on a FarCyte reader (Tecan) equipped with 440 nm excitation and 480 nm emission filters. The samples contained ∼100 µl of the peptide/protein solution in 20 mM Na-phosphate (pH 7.2), 50 mM NaCl, and 10 µM thioflavin T. Plates were sealed with polyolefin tape (Nunc) and incubated at 37°C. Data points were recorded every 5 min with 2 min of linear shaking before the measurement. The experiments were carried out using recombinantly produced Aβ42 with an N-terminal methionine. (0.31 MB TIF) [file pbio.1000334.s003.tif]

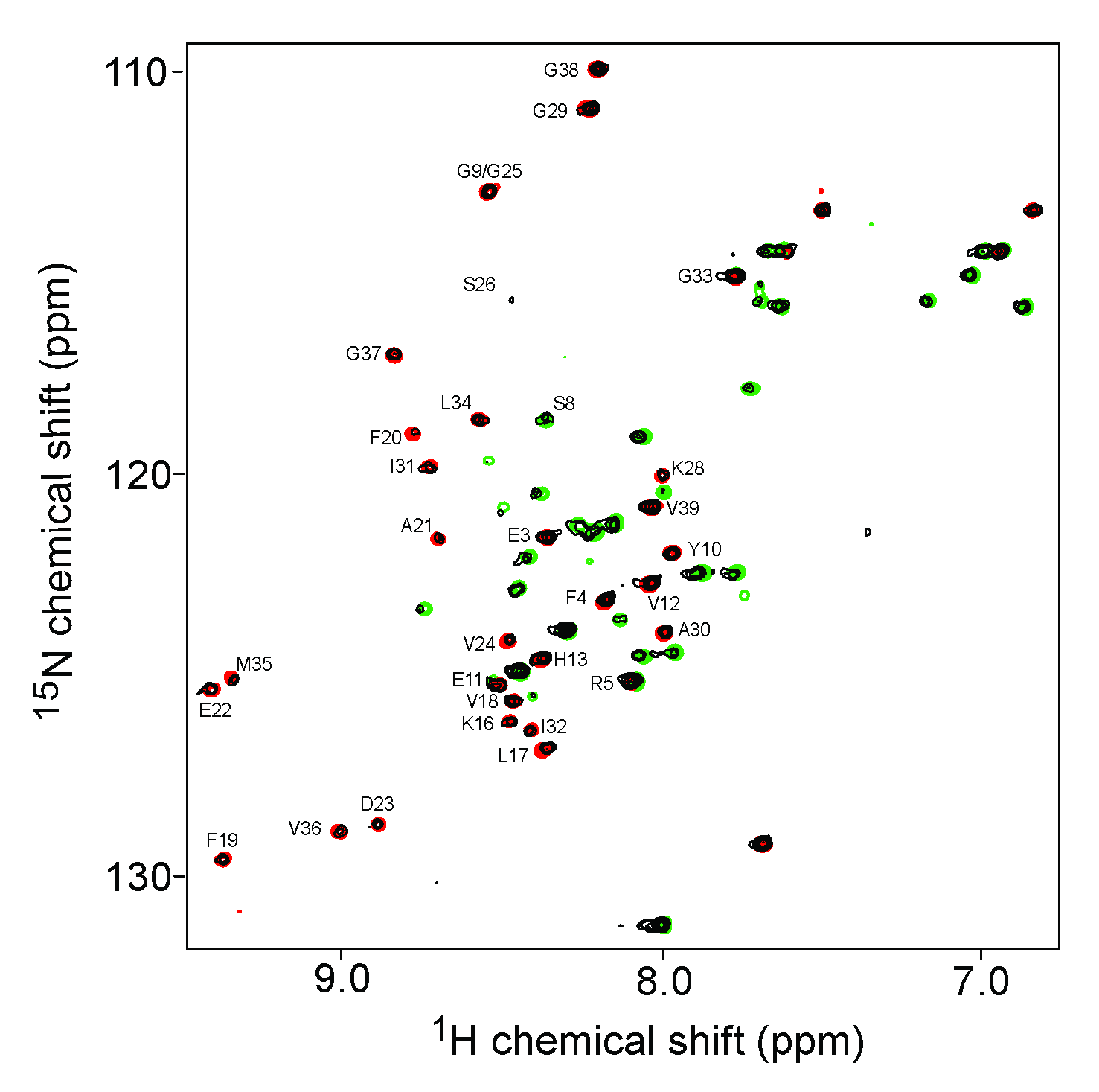

Supplement: Figure S4 — Dissolution of 15N-Aβ40 from fibrils by ZAβ3 monitored by NMR. 15N HSQC NMR spectrum of a fibril dissolution sample (black), starting from 300 µM 15N-Aβ40 in fibrils, recorded during the first 24 h after addition of 325 µM ZAβ3 and 5 µM 15N-ZAβ3. For reference, the spectra of bound Aβ40 (red; assigned) and free ZAβ3 (green) are shown. (The spectrum of fibrillar Aβ40 before ZAβ3 addition shows no resonances at this contour levelling). Buffer, 20 mM sodium phosphate, pH 7.2. Temperature, 37°C. Recombinantly produced Aβ40 with an N-terminal methionine was used. (0.32 MB TIF) [file pbio.1000334.s004.tif]

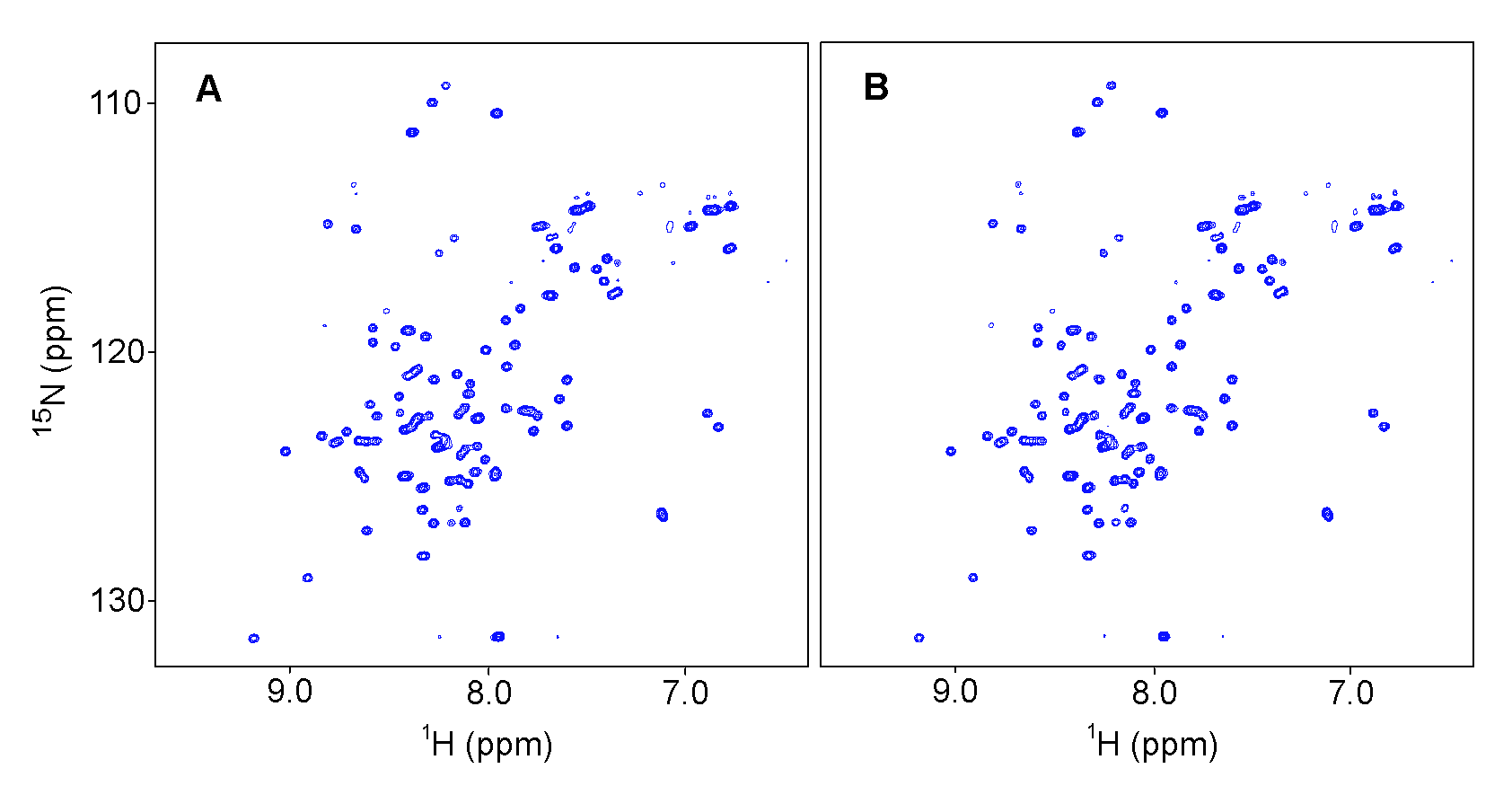

Supplement: Figure S5 — Stability of the Aβ40:ZAβ3 complex in the presence of Aβ40 amyloid fibrils. (A) 15N-HSQC NMR spectrum of 100 µM 15N-ZAβ3 bound to 100 µM unlabeled Aβ40 before addition and (B) after addition of 100 µM 15N-Aβ40 in amyloid fibrils and incubation for 5 days at 37°C. Buffer, 20 mM sodium phosphate, pH 7.2, 0.1% sodium azide. Fibrillar 15N-Aβ40 is not detected by solution NMR because of its large size, for which slow tumbling results in line broadening. The spectrum of 15N-ZAβ3 in the bound state (A) is retained in (B), and resonances of 15N-ZAβ3 in the free state do not appear. This demonstrates that Aβ40 does not leave the complex to be incorporated into the fibrils, i.e. the complex is stable in the presence of Aβ40 amyloid fibrils. Moreover, resonances of 15N-Aβ40 bound to ZAβ3 do not appear in (B), i.e. 15N-Aβ40 monomers do not dissociate from the fibrils to exchange with unlabeled Aβ40 monomers in the ZAβ3 complex. This finding is in agreement with the high kinetic stability of Aβ amyloid fibrils reported in this study. The lifetime of the Aβ40:ZAβ3 complex was determined as 2.6 (±0.3) h at 21°C. Dissociation of the complex cannot therefore be rate-limiting in this experiment. Lifetime determination was carried out by successive recording of the 15N-HSQC NMR spectrum of 15N-ZAβ3:15N-Aβ40 complex after addition of an excess of unlabeled ZAβ3 and monitoring the decrease in the intensity of the resonances assigned to bound 15N-ZAβ3. Recombinantly produced Aβ40 with an N-terminal methionine was used. (0.20 MB TIF) [file pbio.1000334.s005.tif]

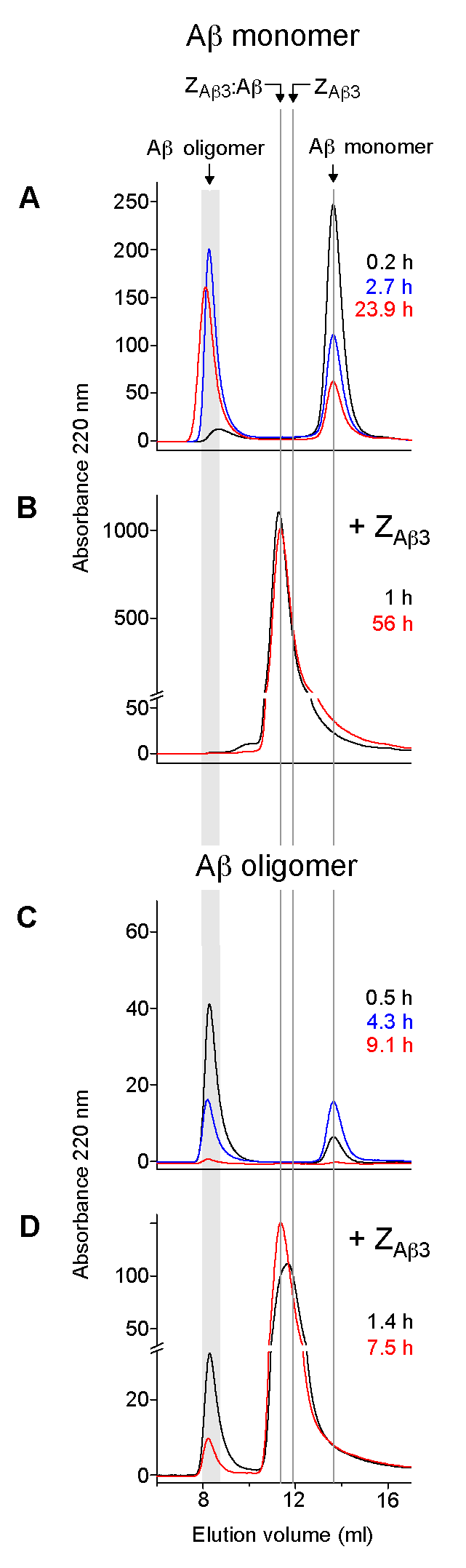

Supplement: Figure S6 — Aβ42 oligomer formation and dissolution analyzed by SEC. Elution volumes of monomeric and oligomeric Aβ42, free ZAβ3 Affibody, and the ZAβ3:Aβ42 complex on a Superdex 75 10/300 column, with a nominal resolution of 3,000 to 70,000 Da, are indicated. Aβ42 oligomers elute at the void volume (8.3 ml) and Aβ42 fibrils cannot enter the column. (A) A solution of 100 µM Aβ42 was incubated without stirring at 20°C. SEC analysis of samples removed at different times reveals the decrease in concentration of monomeric Aβ42 with time and the transient formation of oligomeric species, followed by formation of HMW aggregates (fibrils). (B) Analysis of an equivalent Aβ42 solution also containing a 1.2-fold excess of the ZAβ3 Affibody shows that the ZAβ3:Aβ42 remains stable without oligomer or HMW aggregate formation. (C,D) Oligomer dissolution: isolated oligomer Aβ42 fractions isolated subjected to a second incubation followed by SEC analysis. In the absence of ZAβ3 (C), these dissolve on a timescale of several hours and monomeric Aβ42 appears transiently prior to fibril formation. Oligomer dissolution in the presence of an 1.2-fold excess of ZAβ3 (D) results in ZAβ3:Aβ42 complex formation manifested in a small but significant shift in the elution volume of the ZAβ3 Affibody. Recombinantly produced Aβ42 with an N-terminal methionine was used. (0.18 MB TIF) [file pbio.1000334.s006.tif]

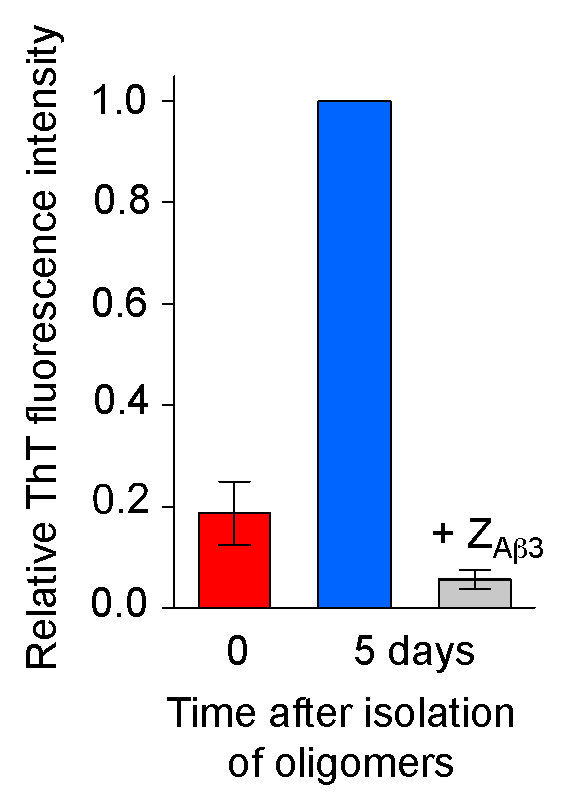

Supplement: Figure S7 — Aβ42 oligomer dissolution analyzed by ThT fluorescence. Aβ42 oligomer fractions were isolated by SEC and incubated at 20°C. The initial fluorescence (red bar) associated with ThT binding to oligomeric Aβ42 increases upon formation of fibrils (blue) or decreases as oligomers dissolve in the presence of an excess of ZAβ3 (grey). ThT fluorescence was recorded on a Varian Cary Eclipse spectrofluorometer at 480 nm, with excitation at 446 nm. Samples were diluted to final Aβ42 concentrations of 1 µM into 20 mM sodium phosphate, 50 mM NaCl, pH 7.2, supplemented with 10 µM ThT. The intensity of the fibril sample was set to unity. Error bars give the estimated standard deviation of four independent oligomer dissolution experiments. Recombinantly produced Aβ42 with an N-terminal methionine was used. (0.09 MB TIF) [file pbio.1000334.s007.tif]
