# Supplementary material for: Sequestration of the Aβ Peptide Prevents Toxicity and Promotes Degradation In Vivo
Source: PLoS Biol. 2010 Mar 16;8(3):e1000334. doi: 10.1371/journal.pbio.1000334 (PMC2838747; doi:10.1371/journal.pbio.1000334)
Supplement: Table S1 — Transgenic fly survival (median life span). (0.05 MB PDF) [file pbio.1000334.s008.pdf]

Table S1. Transgenic fly survival (median life span).

Fly line	Median lifespan in days (± standard error)
w1118 (wild type)	38 (±1.8)
Z domain	40 (±1.3)
$Z_{A\beta 3}$ Affibody	42 (±1.4)
$(Z_{A\beta 3})_2$ Affibody	42 (±2.1)
$A\beta_{40}$	38 (±2.1)
Z domain + $A\beta_{40}$	42 (±3.0)
$Z_{A\beta 3}$ Affibody + $A\beta_{40}$	41 (±2.0)
$(Z_{A\beta 3})_2$ Affibody + $A\beta_{40}$	38 (±2.4)
$A\beta_{42}$	28 (±0.4)
Z domain + $A\beta_{42}$	27 (±0.5)
$Z_{A\beta 3}$ Affibody + $A\beta_{42}$	32 (±0.7)
$(Z_{A\beta 3})_2$ Affibody + $A\beta_{42}$	40 (±1.2)
$A\beta_{42E22G}$	9 (±0.5)
Z domain + $A\beta_{42E22G}$	12 (±0.4)
$Z_{A\beta 3}$ Affibody + $A\beta_{42E22G}$	20 (±0.2)
$(Z_{A\beta 3})_2$ Affibody + $A\beta_{42E22G}$	31 (±0.8)
